# Phase 4 Multinational Multicenter Retrospective and Prospective Real-World Study of Nivolumab in Recurrent and Metastatic Squamous Cell Carcinoma of the Head and Neck

**DOI:** 10.3390/cancers15143552

**Published:** 2023-07-09

**Authors:** Anagha Gogate, Bryan Bennett, Zia Poonja, Grant Stewart, Ana Medina Colmenero, Petr Szturz, Courtney Carrington, Clara Castro, Eric Gemmen, Ashley Lau, Alberto Carral Maseda, Eric Winquist, Virginia Arrazubi, Desiree Hao, Audrey Cook, Joaquina Martinez Galan, Lisardo Ugidos, David Fernández Garay, David Gutierrez Abad, Robert Metcalf

**Affiliations:** 1 Bristol Myers Squibb, Lawrenceville, NJ 08648, USA; 2 Bristol Myers Squibb, Uxbridge UB8 1DG, UK; 3 BC Cancer, University of British Columbia, Victoria, BC V8R 6V5, Canada; 4 Royal Cornwall Hospital NHS Trust, Truro TR1 3LJ, UK; 5 Fundación Centro Oncologico de Galicia, 15009 Coruña, Spain; 6 Department of Oncology, University of Lausanne (UNIL), Lausanne University Hospital (CHUV), 1011 Lausanne, Switzerland; 7 IQVIA, Parsippany-Troy Hills, NJ 07054, USA; 8 IQVIA, 2740-266 Oeiras, Portugal; 9 IQVIA, Falls Church, VA 22042, USA; 10 IQVIA, Kirkland, QC H9H 5M3, Canada; 11 Department of Oncology, Hospital Universitario Lucus Augusti, 27003 Lugo, Spain; 12 Department of Oncology, London Health Sciences Centre, Western University, London, ON N6A 5W9, Canada; 13 Oncology, Instituto de Investigación Sanitaria de Navarra (IdiSNA), Hospital Universitario de Navarra, 31008 Pamplona, Spain; 14 Thoracic and Head & Neck Oncology, Tom Baker Cancer Centre, Cumming School of Medicine, University of Calgary, Calgary, AB T2N 4N2, Canada; 15 Cheltenham General Hospital, Cheltenham GL53 0BG, UK; 16 Instituto de Investigación Biosanitaria, Hospital Universitario Virgen Nieves, 18014 Granada, Spain; 17 Oncology, Hospital Universitario HM Madrid Sanchinarro, 28050 Madrid, Spain; 18 Oncology, Hospital Universitario de Jaén, 23007 Jaén, Spain; 19 Oncology, Hospital Universitario de Fuenlabrada, 28942 Madrid, Spain; 20 The Christie NHS Foundation Trust, Manchester M20 4BX, UK

**Keywords:** nivolumab, head and neck squamous cell carcinoma, recurrent/metastatic head and neck cancer, real-world data, prospective study, retrospective study, patient-reported outcomes, SCCHN, HNSCC

## Abstract

**Simple Summary:**

In 2020, there were 880,000 new cases and 440,000 deaths attributed to head and neck cancer worldwide. Nivolumab was approved in 2016 in the United States, and 2017 in the European Union and Canada, for use in patients with recurrent/metastatic squamous cell carcinoma of the head and neck with progressive disease at or within 6 months after platinum-based therapy. This study was conducted to capture the real-world utilization of nivolumab and to assess the impact of treatment on quality of life among affected patients. The retrospective protocol (VOLUME) described the effectiveness of treatment with nivolumab in terms of survival times and response on imaging, while the prospective protocol (VOLUME-PRO) described the health-related quality of life (HRQoL) among patients treated with nivolumab.

**Abstract:**

This study examined the real-world use of nivolumab in patients with recurrent/metastatic squamous cell carcinoma of the head and neck (R/M SCCHN). This was a multinational retrospective study (VOLUME) assessing treatment effectiveness and safety outcomes and a prospective study (VOLUME-PRO) assessing HRQoL and patient-reported symptoms. There were 447 and 51 patients in VOLUME and VOLUME-PRO, respectively. Across both studies, the median age was 64.0 years, 80.9% were male, and 52.6% were former smokers. Clinical outcomes of interest included real-world overall survival (rwOS) and real-world progression-free survival (rwPFS). The median rwOS was 9.2 months. Among patients with at least one assessment, 21.7% reported their best response as ‘partial response’, with 3.9% reporting ‘complete response’. The median duration of response (DoR) and median rwPFS were 11.0 months and 3.9 months, respectively. At baseline, VOLUME-PRO patients reported difficulties relating to fatigue, physical and sexual functioning, dyspnea, nausea, sticky saliva, dry mouth, pain/discomfort, mobility, and financial difficulties. There were improvements in social functioning and financial difficulties throughout the study; however, no other clinically meaningful changes were noted. No new safety concerns were identified. This real-world, multinational, multicenter, retrospective and prospective study supports the effectiveness and safety of nivolumab for R/M SCCHN patients.

## 1. Introduction

Squamous cell carcinoma of the head and neck (SCCHN) represents 90% of head and neck cancers, and is the sixth most commonly diagnosed malignancy globally [1]. The public health impact of this condition is of significant concern and continues to grow, with 880,000 new cases and 440,000 deaths worldwide in 2020 [1]. Patients with SCCHN tend to present with a loco-regionally advanced stage, and more than half of patients’ tumors will relapse locally or metastasize to distant sites. Recurrent/Metastatic (R/M) SCCHN has limited treatment options and an overall poor prognosis [2,3,4,5]. Patients with R/M SCCHN also have poor health-related quality of life (HRQoL) as a result of physical disease-related symptoms, treatment effects, and long-term mental and social outcomes related to living with an incurable condition [6].

Nivolumab, an immunoglobulin monoclonal antibody, was approved in 2016 in the United States and 2017 in the European Union and Canada for use in patients with R/M SCCHN with progressive disease at or within 6 months after platinum-based therapy [7,8,9]. CheckMate 141 was a randomized Phase 3 trial which investigated whether overall survival (OS) could be improved in cisplatin-refractory R/M SCCHN patients with nivolumab compared with the investigator’s choice standard of care monotherapy (methotrexate, docetaxel, or cetuximab). Nivolumab demonstrated a significantly longer OS compared with standard therapy, with a median OS of 7.5 months (95% confidence inteval [CI], 5.5 to 9.1) versus 5.1 months (95% CI, 4.0–6.0) in the standard therapy group [10]. The survival benefit was maintained at 2 years; nivolumab-treated patients had a 24-month OS rate of 16.9% (95% CI, 12.4–22.0) compared to 6% (95% CI, 2.7–11.3) for investi-gator’s choice [4].

CheckMate 141 also assessed HRQoL and found that nivolumab stabilized symptoms and functioning compared to the investigator’s choice, for which clinically meaningful deterioration from baseline to weeks 9 and 15 was reported [6]. Nivolumab also delayed the time to deterioration in global health status and other HRQoL outcomes compared to the investigator’s choice.

The current study was conducted to determine the outcomes following the real-world utilization of nivolumab among R/M SCCHN patients across several countries, including Canada, Spain, Swiaerland, and the United Kingdom (UK). The retrospective protocol (VOLUME [NiVOLUMab in the Real-World in Patients with Squamous Cell Carcinoma of the Head and Neck]) aimed to describe the effectiveness associated with treat ment with nivolumab while the prospective protocol (VOLUME-PRO [Patient-Reported Outcomes]) aimed to describe the HRQoL and patient-reported symptoms among patients treated with nivolumab. Both protocols reported the incidence of safety events or medically important occurrences while on nivolumab treatment.

## 2. Materials and Methods

### 2.1. Study Design

VOLUME was a retrospective, multinational, multicenter, Phase 4 study designed to observe the utilization of nivolumab in the real-world setting among R/M SCCHN patients. The separate and complimentary study, VOLUME-PRO, aimed to assess HRQoL where data were available. All patients being treated at 23 participating health care institutions with a diagnosis of R/M SCCHN in the UK, Swiaerland, Spain, or Canada were eligible for participation in these studies. Patients were required to have been prescribed nivolumab by a physician in an oncologist’s office, hospital, or infusion center at the time of enrollment. Additionally, those enrolled in VOLUME-PRO were required to complete the following 5 PRO assessments at baseline/enrollment and again approximately 6–8 weeks later at the standard of care (SOC) visit: HRQoL and symptom assessment using the European Organisation for Research and Treatment of Cancer (EORTC) QLQ-C30, EORTC QLQ-H&N35, and the 5-Level EQ-5D; patient satisfaction with treatment using the Cancer Therapy Satisfaction Questionnaire (CTSQ); and patient-reported general health and impact of symptom severity on work productivity and regular activities through the Work Productivity and Activity Impairment Questionnaire: General Health (WPAI: GH) V2.0.

VOLUME data were collected beginning at 6 months prior to nivolumab initiation (baseline) and continuing up to 36 months following nivolumab initiation. Data for VOLUME-PRO patients were collected beginning at enrollment (baseline) and again at the SOC visit.

Given the non-interventional nature of both studies, all treatment decisions and the frequency of clinical assessments were performed according to routine clinical practice, and no visits were mandated during either study.

### 2.2. Eligibility Criteria

Patients 18 years of age and older with a diagnosis of R/M SCCHN and documented evidence of nivolumab treatment for R/M SCCHN were eligible for participation in both VOLUME and VOLUME-PRO. Additionally, patients who provided consent and were willing to self-complete on-site PRO measures were eligible for participation in VOLUMEPRO. Patients who were concurrently enrolled in an interventional SCCHN clinical trial and patients who received systemic treatment for any other primary cancer within 6 months of enrollment were not eligible for participation in either study.

### 2.3. Study Variables

#### 2.3.1. Independent Variables

Patients’ medical records were used to obtain independent variables, which included patient demographics (birthdate, sex, smoking status), disease characteristics (tumor and metastasis location, histology), comorbidities, tumor biomarkers (programmed death-ligand 1 [PD-L1] status and human papilloma virus [HPV] p16 status), previous treatment history, and performance status (Eastern Cooperative Oncology Group [ECOG] performance scale). PD-L1 is reported using the tumor proportion score (TPS), which represents the percentage of positive tumor cells with partial or complete staining [11]. Medical records were also extracted for nivolumab treatment details, including exposure to nivolumab, reason for treatment discontinuation, subsequent treatment, and response to treatment.

#### 2.3.2. Endpoints

##### Clinical Effectiveness Measures

Clinical effectiveness measures were assessed by several study endpoints of interest including real-world OS (rwOS), clinical response, response rate, and real-world progression-free survival (rwPFS). The primary endpoint for VOLUME was rwOS, defined as the time from index date (initiation of treatment with nivolumab) until date of death from any cause. RwOS was censored on the last date a subject was known to be alive, defined as their final visit captured in their medical records during the study period. For patients who were lost to follow-up or who enrolled in a clinical trial, data were censored at that point but all previous records were retained to allow analysis of person-time, a measurement that estimates the time at risk as defined by the amount of follow-up time patients contributed to the study.

Clinical responses, based on physician assessment, were classified as complete response, partial response, stable disease, progressive disease, or non-evaluable. Response assessments were collected based on methods considered part of standard clinical practice, which included computed tomography (CT) scans, magnetic resonance imaging (MRI), ultrasounds, and other similar methods. Duration of response (DoR) was defined as the time from documentation of tumor response to the date of acknowledged progressive disease, as recorded in patient charts through the 36-month post-index date. Real-world response rate was defined as the proportion of nivolumab-treated patients with a complete or partial response based on physician assessment, as described in medical charts. Response rate percentages were based on the number of patients with at least one tumor assessment during the follow-up period.

RwPFS was defined as time since index date until the first date of documented progressive disease (as assessed by the physician), date of death from any cause, end of postindex period, or censored at date of last follow-up, whichever occurred first.

##### Nivolumab Treatment Measures

Duration of treatment was defined as the time (months) from index date to permanent drug discontinuation due to any cause (including death). Time-to-event analysis was performed on the duration of treatment to take into consideration any events occurring after treatment onset that led to an interruption in the treatment that would not occur otherwise (e.g., death). Patients not permanently discontinuing nivolumab were censored at the end of follow-up period, or at the date of loss to follow-up, whichever occurred first.

Permanent drug discontinuation was defined as no infusion 6 weeks or more after the last infusion if the dose given was recorded as 3 mg/kg or 240 mg every 2 weeks, or 9 weeks or more if the dose given was recorded as 480 mg every 4 weeks. If nivolumab was restarted after the time period mentioned above, it was considered as part of a new treatment line.

#### 2.3.3. Patient-Reported Outcome Measures

The following self-administered PRO measures were collected from VOLUME-PRO participants.

The European Organisation for Research and Treatment of Cancer Quality of Life Questionnaire (EORTC QLQ-30) was developed to assess HRQoL in patients with cancer and measures the following domains: global health status/QoL, functional scales (physical, role, emotional, cognitive, and social functioning), and symptom scales. Responses for each item, except for global health status/QoL, are recorded on a 4-point Likert scale ranging from 1 (“Not at all”) to 4 (“Very much”). Global health status/QoL is recorded on a scale from 1 (very poor) to 7 (excellent). All the scales and single-item measures range in score from 0 to 100 with higher scores indicating a better HRQoL for functional scales and global health status, and lower scores for symptom scales representing a better HRQoL [12]. A score change of 5–10 has been regarded as the range for minimal clinically important difference [13].

The EORTC QLQ-H&N35 is a tumor-specific module used in conjunction with the EORTC QLQ-C30 and contains 7 domains: pain, swallowing, senses, speech, social eating, social contact, sexuality, and 11 other single items [14]. Scoring is based on a 4-point Likert scale ranging from 1 (“Not at all”) to 4 (“Very much”) and some binary (yes/no) items. Scores by both dimension and items range from 0 to 100, in which lower scores represent a better HRQoL [12]. A score change of 10–20 indicates a medium to a large change in functional and symptom status, based on standardized response mean values [15,16]. For this module, a score change of 9.43 is considered a minimal clinically important difference in HRQoL [17].

The 5-Level EQ-5D (EQ-5D-5L) describes health status and functioning and consists of a descriptive system and a visual analog scale (VAS) called the EQ-VAS [18,19]. The descriptive system includes 5 dimensions, mobility, self-care, usual activities, pain/discomfort, and anxiety/depression, in which patients record a response using a scale ranging from 1 (representing no problems) to 5 (representing extreme problems). An index score is then generated between 0 and 1, with higher scores indicating better HRQoL. Estimates of the minimally important difference for the index score are between 0.037 and 0.069. The EQ-VAS is a self-rated, vertical scale where the endpoints are labeled ‘Best imaginable health state’ and ‘Worst imaginable health state’, with numeric values of 100 and 0, respectively [20]. Minimally important differences for EQ-VAS scores are reported to range from 7 to 12 [21].

The CTSQ was developed to assess satisfaction and preference for chemotherapy and/or biological therapy among patients with cancer. This 16-item questionnaire assesses 3 dimensions, expectations of therapy, feelings about side effects, and satisfaction with therapy, using a 5-point Likert scale. Scores by dimension range between 0 and 100, with a higher score representing better satisfaction. A score change of 0.5 standard deviation (SD) in the dimension is regarded as clinically meaningful [22].

The WPAI: GH V2.0 was developed to measure the impact on work productivity and regular activities attributable to a specific health problem [23]. The WPAI: GH V2.0 assesses absenteeism (the loss of productivity caused by being absent from work because of poor health), presenteeism (the impact on productivity whilst at work because of health problems), work productivity loss, and activity impairment [24]. Response options include yes/no and a 0–10 numerical rating scale. Scores are calculated as impairment percentages, and higher numbers indicate greater impairment and less productivity. A score change of 7% for the WPAI: GH V2.0 is regarded as clinically meaningful [25].

PRO Measures were translated into Spanish, French, and German based on patient location and preference.

### 2.4. Statistical Considerations

All analyses were exploratory and descriptive in nature. The studies were not designed to support any formal statistical testing, and therefore no formal sample size/statistical power calculations were performed. There were no comparative analyses performed between the VOLUME and VOLUME-PRO studies.

Descriptive analysis of the data was performed using summary statistics for categorical and continuous variables. Frequency tables were generated for categorical data. Continuous variables and percentages were computed based on the number of non-missing data in the denominator. Changes in PRO scores were assessed using clinically meaningful changes in mean score, as well as statistically using a Wilcoxon non-parametric paired *t*-test.

Analyses of rwOS and rwPFS were conducted using Kaplan–Meier (KM) methods and included a summary of the number and percentage of censored patients. All statistical analyses were performed using SAS® (SAS Institute, Cary, NC, USA), Version 9.4 or higher.

## 3. Results

There were 447 patients enrolled in VOLUME, 51 patients in VOLUME-PRO, and 17 enrolled in both studies. There were 23 and 17 sites that enrolled patients in VOLUME and VOLUME-PRO, respectively.

### 3.1. Demographics and Clinical Characteristics

Similar distributions of demographics and clinical characteristics were found between patients in VOLUME and VOLUME-PRO. Patient characteristics are summarized in Table 1.

The overall participant age ranged from 32 to 89 with a median (interquartile range [IQR]) age of 64.0 (12.0) years. More than 80% of patients were male. Approximately half (*n* = 233, 52.6%) reported being a former smoker and 117 (26.4%) reported being current smokers. Over half of patients reported having an ECOG performance status of 1 (restricted in physically strenuous activities) (*n* = 218, 58.1%).

At baseline (defined as ≤6 months from the start of nivolumab treatment for VOLUME patients, and at study enrollment for VOLUME-PRO patients), the most common primary tumor locations were the oropharynx (*n* = 166, 34.6%), oral cavity (*n* = 132, 27.5%), and larynx (*n* = 73, 15.2%). Over one-third of patients (*n* = 162, 35.7%) had an R/M SCCHN diagnosis with a T4 tumor size, classified by the American Academy of Otolaryngology—Head and Neck Surgery Foundation as a tumor with evidence of spread beyond the site of origin. Slightly fewer than half of patients (*n* = 205, 44.5%) reported an N2 nodal involvement indicating some lymph node metastasis [26]. More than half (*n* = 236, 53.4%) of patients had distant dissemination (M1), with the most common location of metastasis being the lungs (*n* = 217, 46.5%).

Among 67 VOLUME patients with recorded PD-L1 tumor proportion scores (TPS) prior to starting nivolumab, the median (IQR) TPS percent expression was 5.0 (34.0)%. Of the 14 patients in VOLUME-PRO with PD-L1 expression status prior to nivolumab, the median (IQR) TPS percent expression was 1.5 (4.0)%. Approximately half of VOLUME and VOLUME-PRO patients that had a recorded assessment of their HPV p16 status (*N* = 218) were p16 positive (*N* = 114, 52.3%).

Among 447 VOLUME patients, 404 (90.4%) received a prior or concomitant anti-cancer therapy for SCCHN, irrespective of stage. There were 269 (72.1%) patients with one prior line of therapy. The majority of patients (*n* = 377, 84.3%) received prior or concomitant cytotoxic drugs, about half *(n* = 229, 51.2%) received radiotherapy, and 88 (19.7%) received surgery. Among those patients who received prior or concomitant cytotoxic drugs, approximately half (*n* = 194, 51.5%) received cisplatin, 193 (51.2%) patients reported taking carboplatin, and one-third (*n* = 125, 33.2%) of patients received 5-fluorouracil. Among 51 VOLUME-PRO patients, 37 (72.5%) received a prior or concomitant anti-cancer therapy for SCCHN. Twenty-seven (87.1%) reported one line of therapy. Thirty-two (62.7%) received prior or concomitant cytotoxic drugs, eighteen (35.3%) received radiotherapy, and ten (19.6%) received surgery. Like patients in VOLUME, VOLUME-PRO patients who received cytotoxic drugs most commonly reported taking carboplatin (*n* = 16, 50%), cisplatin (*n* = 14, 43.8%), and 5-fluorouracil (*n* = 11, 34.4%). It is possible that some patients received cytotoxic drugs under a combination therapy regimen; however, prior cytotoxic data were not collected under the context of combination therapy per protocol.

### 3.2. Nivolumab Exposure

There were 434 (97.1%) VOLUME patients that permanently discontinued nivolumab due to any cause including death. The median time to discontinuation was 3.2 months and the median (IQR) dose among VOLUME patients was 240.0 (36.0) mg. Patients reported either one or two nivolumab treatment lines, with the median being one line. Nivolumab exposure is summarized in Table 2.

### 3.3. VOLUME

#### 3.3.1. Real-World Overall Survival

**Figure 1:**
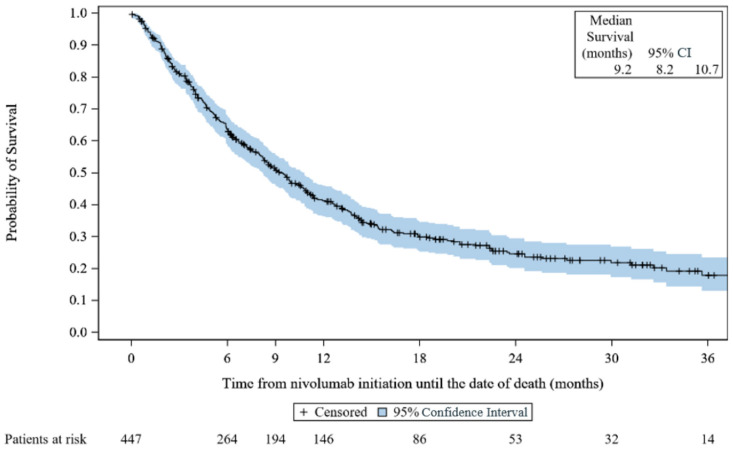
Kaplan–Meier survival curves for real-world overall survival: VOLUME. CI: confidence interval.

At the end of the study, 301 (67.3%) deaths occurred. The median rwOS was 9.2 months (95% CI, 8.2 to 10.7) (Figure 1). The rwOS rate was 63.7% (95% CI, 58.9 to 68.1) at 6 months, 51.0% (95% CI, 46.1 to 55.7) at 9 months, 41.2% (95% CI, 36.4 to 46.0) at 1 year, 24.6% (95% CI, 20.1 to 29.4) at 2 years, and 17.9% (95% CI, 13.0 to 23.5) at 3 years.

#### 3.3.2. Response to Treatment

**Figure 2:**
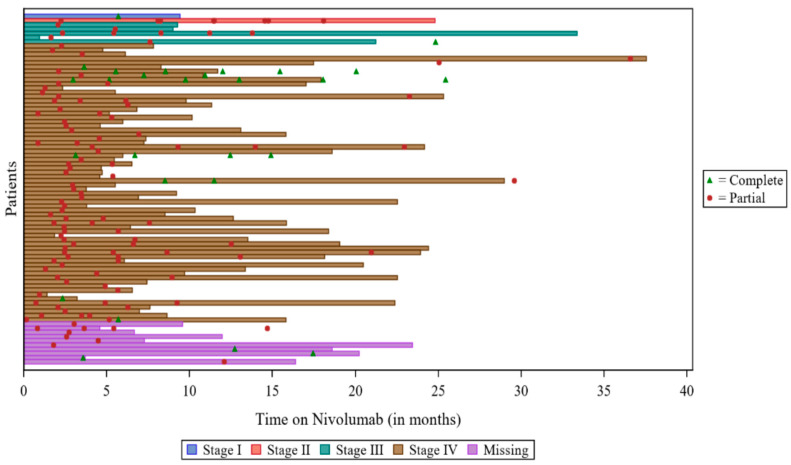
Swimmers plot for responders: VOLUME. Note: There were 2 patients with “partial response”, but the date of response and the date of assessment were both missing, so these patients were not included in the plot. There was 1 patient with a response, but the type of response was missing, so the patient was not included in the plot. For 2 patients, the date of Nivolumab discontinuation was not available, so the date of study discontinuation was used.

Among 334 patients with at least one assessment, 85 (25.4%) assessments reported a response to treatment. Seventy-two (21.7%) patients reported ‘partial response’ as their best response, and 13 (3.9%) reported ‘complete response’ as their best response. Over half (n = 185, 55.7%) reported ‘disease progression’ as their best response, and 60 (18.1%) reported ‘stable disease’. Two patients (0.6%) had non-evaluable responses, and two (0.6%) patients had missing responses. The swimmers plot depicting response is provided in Figure 2, below.

#### 3.3.3. Time to Response and Response Rate

Among patients with a response to treatment, the majority of responses were reported between baseline and Month 3 (n = 52) or Month 3 to Month 6 (n = 41) and had response rates of 23.0% (95% CI, 17.7 to 29.1) and 28.5% (95% CI, 21.3 to 36.6), respectively.

#### 3.3.4. Duration of Response (DoR)

Among 85 patients with treatment responses, the median DoR was 11.0 months (95% CI, 8.3 to 22.1) with a range (with censored values) of 0.5–41.3 months. Approximately half (47.9%, 95% CI, 36.2 to 58.6) of the patients had ongoing response at 1 year, and 31.5% (95% CI, 19.1 to 44.6) at 2 and 3 years. Overall, 47 (55.3%) patients who had a response experienced progressive disease or death.

#### 3.3.5. Real-World Progression-Free Survival

**Figure 3:**
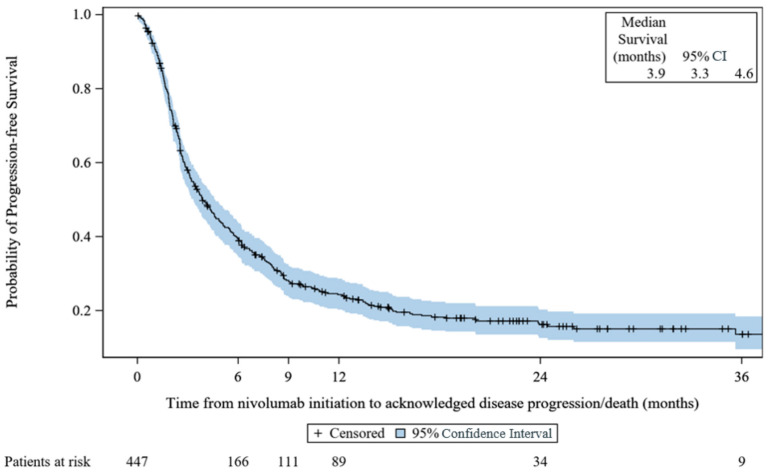
Kaplan–Meier survival curves for progression-free survival: VOLUME. CI: confidence interval.

There were 351 (78.5%) patients with reported progressive disease or death. The median rwPFS was 3.9 months (95% CI, 3.3 to 4.6) with a range (including censored values) of 0.0–51.3 months. The KM rwPFS curve is provided in Figure 3, below.

### 3.4. VOLUME-PRO

#### 3.4.1. Summary of Patient-Reported Outcome Measures

A full summary of PRO scores is presented in Table 3. Completion rates ranged between 96.1% and 98.0% at enrollment and between 80.4% and 86.3% at the SOC visit across all measures.

The EORTC QLQ-C30 results showed that among the functioning scales, cognitive functioning scores had the highest mean (SD) scores, and therefore a better HRQoL, at both the enrollment and the SOC visit (82.7 [19.33] and 84.9 [17.58], respectively). The role function scores had the lowest mean (SD) scores of all domains at both the enrollment and the SOC visits (67.7 [33.91] and 68.3 [34.49], respectively), followed by social functioning at enrollment (70.3 [31.82]) and physical functioning at the SOC visit (69.7 [26.18]), indicating lower HRQoL related to these measures. Lower scores for symptom scales of the EORTC QLQ-30 indicate better HRQoL. Among symptom scales, diarrhea had the lowest mean (SD) score at enrollment (9.3 [22.38]) and financial difficulties (11.4 [23.11]) at the SOC visit. Fatigue had the highest mean (SD) score at both enrollment and the SOC visit (39.3 [28.32] and 39.9 [30.36], respectively). The mean (SD) global health status/QoL scores at enrollment and the SOC visit were 64.2 (24.24) and 61.9 (21.32), respectively, in which higher scores indicate better HRQoL.

Similar to the EORTC QLQ-30, higher scores indicate a better HRQoL for functional scales of the EORTC QLQ-H&N35. Lower scores for symptom scales represent a better HRQoL. For the EORTC QLQ-H&N35, responses indicate that social contact had the lowest mean score at both the enrollment and the SOC visit, among the seven domains, as indicated by means (SD) of 22.2 (28.25) and 18.8 (26.23), respectively. The highest mean score was sexuality at both the enrollment and the SOC visit, as indicated by the means (SD) of 34.8 (39.70) and 40.4 (38.09), respectively. Among the 11 individual item scales, sticky saliva had the highest mean score at both the enrollment and the SOC visit, 48.7 (36.40) and 39.8 (40.29), respectively. The lowest mean score at enrollment was weight, 7.3 (13.95), and feeding tube score at the SOC visit, 6.3 (13.25), among the 11 individual items. The overall EQ-5D-5L index mean (SD) score at enrollment was 0.8 (0.21) and 0.8 (0.23) at the SOC visit. For this measure, values closer to 1 are indicative of better health. For the pain/discomfort dimension, 16 (31.4%) patients reported having no pain, while 14 (27.5%) and 18 (35.3%) patients overall reported having slight or moderate pain/discom fort at enrollment, respectively. At the SOC visit, 10 (19.6%) patients reported having no pain, with 16 (31.4%) and 13 (25.5%) reporting slight or moderate pain or discomfort, respectively. For the mobility dimension, 22 (43.1%) patients reported having no problems in walking about at enrollment, decreasing to 16 (31.4%) patients at the SOC visit. Lastly, data for the usual activities dimension showed that at enrollment approximately half (*n* = 23, 45.1%) of patients reported having no problems doing usual activities, which decreased slightly to 21 (41.2%) patients at the SOC visit (Supplementary Figure S1).

The mean (SD) EQ-5D VAS score at enrollment was 67.9 (25.13), and 70.1 (22.0) at the SOC visit. Given the VAS score endpoints are labeled ‘Best imaginable health state’ and ‘Worst imaginable health state’, with numeric values of 100 and 0, respectively, our study demonstrates that our patients had a better health state at the follow-up visit.

The highest mean (SD) score of the CSTQ was expectations of therapy at enrollment (68.3 [23.90]), and side effects at the SOC visit (64.7 [14.53]). Satisfaction with therapy domain scores had the lowest mean score at both the enrollment and the SOC visit: 37.6 (10.67) and 37.1 (13.15), respectively. Of note, higher CTSQ scores indicate better satisfaction.

Lastly, four (7.8%) patients reported that they were currently in paid employment at enrollment and the SOC visit, as determined by the WPAI: GH V2.0 measure. WPAI scores are calculated as impairment percentages, and higher numbers indicate greater impairment and lower productivity (i.e., worse outcomes). The highest mean (SD) score was work productivity loss, which was 100.0 (0.00) at both enrollment and the SOC visit. The lowest mean (SD) score was presenteeism score, which was 6.7 (11.55) and 0.0 (0.00) at enrollment and the SOC visit, respectively.

#### 3.4.2. Changes in Patient-Reported Outcome Measures

There was a clinically meaningful change in the social functioning and financial difficulties score in the EORTC QLQ-C30. The absenteeism score from the WPAI: GH V2.0 indicated 25% greater impairment; however, this result was derived from only four patient responses and had a wide confidence interval. Aside from these results, there were no other clinically meaningful changes in scores reported for any other PRO measure. Using the Wilcoxon paired test to assess differences in PRO measures, there were no statistically significant changes in mean PRO scores between the enrollment and the SOC visit (Table S4). However, most patients who remained on treatment reported stability or numerical improvements across all the individual domains within PROs (between 6 and 8 weeks).

### 3.5. Safety

#### 3.5.1. VOLUME

Medically important events of any cause occurred in 30.4% (*n* = 136) of VOLUME patients. Commonly reported events were death while on nivolumab treatment (*n* = 54, 12.1%) and physician-perceived lack of effectiveness (*n* = 42, 9.4%). Due to the retrospective study design, reasons for death are not available and therefore the relationship between nivolumab treatment and death cannot be estimated. ‘Other’ medically important events were reported by 13.4% (*n* = 60) of VOLUME patients, which included events such as nivolumab-induced type 1 diabetes, diarrhea, pneumonitis, and fatigue.

#### 3.5.2. VOLUME-PRO

Among 51 VOLUME-PRO patients, 26 (51.0%) patients reported an adverse event (AE) with an incidence rate of 143.63 per 100 patient years. Diarrhea and pruritus were the most frequently reported AEs (*n* = 3, 5.9%); all other AEs were reported among one (2.0%) or two (3.9%) patients. It was reported that seven (13.7%) patients experienced a serious adverse event (SAE). SAEs reported included vomiting, weight loss, hospitalization due to pneumonitis, death due to progressive swallowing dysfunction, palpitations, confused state, and pneumonia aspiration. No instances of death due to nivolumab treatment were recorded among VOLUME-PRO patients.

## 4. Discussion

### 4.1. Key Results

This study examined data from 447 retrospective VOLUME patients and 51 prospective VOLUME-PRO patients. The results from both clinical outcome measures of the VOLUME study and PRO measures of the VOLUME-PRO study were consistent with the evidence generated from clinical trials. Of note, it is important to acknowledge the fundamental differences in the study design, sample selection, and length of follow-up in the VOLUME-PRO cohort compared to the VOLUME cohort. The results identified from these studies are not meant to be directly comparable for those reasons and should be considered as complementary to each other.

The median rwOS observed was consistent with the CheckMate 141 study (7.5 months) and the rwOS of real-world studies conducted in the United States (*n* = 368) and Japan (*n* = 256) [10,27,28]. This is particularly meaningful because, compared to the CheckMate 141 study, the VOLUME patients were older, less fit, and had more comorbidities. In our study, 25.4% of patients with at least one assessment reported a response compared to CheckMate-141, which reported a 13.3% response rate among all patients [10]. It is important to note that the method of assessing response is not standardized in our study and is collected based on physician assessment, due to the retrospective nature of VOLUME. Additional stratified analyses have also demonstrated that the rwOS results are consistent among older adults, as well. Patients who were ≤65 years (*n* = 263, 58.8%) old had a median rwOS of 9.6 months compared to 8.6 months in patients >65 years old (*n* = 184, 41.2%) (Table S2). The older adult population is often underrepresented in clinical trials, and real-world studies can help elucidate gaps in research to improve understanding. Results were consistent with previous studies that have demonstrated Nivolumab to maintain effectiveness in the older adult population [29,30,31,32]. Additionally, rwOS by ECOG performance status was conducted, which demonstrated similar results. The longest median rwOS was among patients with a status of 0 at 12.1 months (95% CI: 8.5, 22.3), followed by patients with an ECOG of 1 (8.4 [95% CI: 6.2, 10.0] months) and the patients with a status of 2 (6.9 [95% CI: 3.5, 9.8] months) (Table S3). The CheckMate-141 study did not include patients with ECOG > 1 [10], so this real-world approach demonstrates that improved rwOS can be seen even among patients with poorer function.

Our median rwPFS of 3.9 months was numerically longer than in the CheckMate 141 study (2.0 months), and in alignment with a retrospective real-world study conducted in the UK (3.9 months) [10,33]. Slightly higher PFS is expected in a real-world study when compared to clinical trials, as patients are likely not receiving follow-up care as frequently as patients in a trial and there may be a lack of standardized follow-up care intervals across study sites. PFS can also vary by investigator, as some may classify progressive disease differently than others. Nevertheless, the study results demonstrate the effectiveness of nivolumab in improving the clinical outcomes of R/M SCCHN patients in a real-world setting.

Our study determined a median dose of 240.0 mg received by VOLUME patients to be aligned with the range of standard dosing of nivolumab. The majority of VOLUME patients discontinued within 3 months of nivolumab starting. There were 31.3% of patients that reported receiving anti-cancer therapies following nivolumab, which was similar to patients in the CheckMate 141 study [34]. The use of cytotoxic drugs was the most commonly reported anti-cancer therapy used following nivolumab (74.3%), with paclitaxel (61.5%) being the most common cytotoxic drug, followed by carboplatin (25%) and cetuximab (23.1%) (Table S1). Permanent discontinuations among this population were frequent, and likely due to progressive disease, death, or the completion of the treatment regimen.

#### 4.1.1. EORTC QLQ-C30

VOLUME-PRO patients reported significant difficulties with physical functioning, fatigue, pain, dyspnea, nausea, and financial difficulties at enrollment, and all exceeded the threshold for clinical importance. These domains are known to be impacted by SCCHN symptoms and effects of treatment [35]. Cognitive functioning had the highest mean scores at both enrollment and the SOC visit. Although role functioning score, which measures a patient’s ability to perform daily activities within both occupational and social roles, had the lowest mean score among VOLUME-PRO patients, this was not noted as meeting the threshold for clinical importance [36].

Social functioning and financial difficulties met the criteria for minimal clinically important difference, and both saw an improvement between the enrollment and follow-up visit. Improved social functioning may be related to reduced symptom effects, allowing patients to participate in social activities more frequently. Financial burden may be linked to out-of-pocket healthcare costs, as well as other external financial challenges [37]. Financial improvement may also be related to better symptom management, which incurs fewer healthcare-related costs. Despite the observed improvement, additional confirmatory research is warranted to show the longevity of the results given that the length of follow-up time in our study was quite limited.

#### 4.1.2. EORTC QLQ H&N35

Results of the EORTC QLQ-H&N35 identified sexuality at both enrollment and the SOC visit as the most problematic domain. In VOLUME-PRO patients, the impact of head and neck cancer on this domain is important and likely multifactorial due to treatment effects such as physical disfigurement, body image difficulties, functional difficulties related to disease or treatment, and impact on mental health [38]. Impaired sexuality has been associated with poorer HRQoL and overall health. A literature review on this topic identified that tools assessing sexuality in patients with head and neck cancer are lacking, and it is important to provide adequate support for patients [39].

Among the 11 individual item scores included in the EORTC QLQ-H&N35, sticky saliva and dry mouth were the two symptoms that were the most bothersome. These are known side effects of head and neck cancer treatment due to radiotherapy with or without chemotherapy, and were consistent with results reported by Bjordal et al. (2000), where recurrent disease patients generally had worse EORTC QLQ-H&N35 scores across domains after radiotherapy [16]. However, Bjordal and colleagues also reported improvements in EORTC QLQ-H&N35 scores over their 5-year follow-up period over time, suggesting that the true direction of our results may not be well understood given our study’s limited follow-up time.

#### 4.1.3. EQ-5D-5L

Results of the EQ-5D-5L demonstrate that VOLUME-PRO patients were largely impacted by pain/discomfort. More than half of patients reported slight or moderate pain at both enrollment and the SOC visit. This was expected, as pain is a common side effect of SCCHN, which can be due to the location of the primary tumor or treatment side effects. Further, pain in the head and neck area can also impact patients’ ability to eat, drink, and speak, likely contributing to poorer HRQoL [40].

Impacts on mobility, ability to do usual activities, and anxiety/depression were also identified. Trends suggesting a worsening of mobility and the ability to execute daily tasks over time were observed. Results of the EQ-5D-VAS showed mean scores of 67.9 and 70.1 at the enrollment and SOC visit, respectively, with scores closer to 100 indicating the best imaginable health state. Our results indicate that patients treated with nivolumab reported a positive perception of their health at these two timepoints.

#### 4.1.4. CTSQ

The results of the CTSQ revealed mean “satisfaction with therapy” domain scores among VOLUME-PRO patients of 37.6 and 37.1 at enrollment and SOC visit, respectively. It is of interest that “feelings about side effects” and “expectations of therapy” at enrollment and the SOC visit were higher (means of 60.6 and 64.7, and 68.3 and 63.1, respectively). The results of these two measures specifically suggest low levels of treatment satisfaction among this population despite higher hopes for therapy impact. While patient satisfaction scores were low, satisfaction with treatment is a complex measure that is often impacted by advanced disease stage, prognosis, and adverse events. It may also be linked to patients’ expectations of remission [41]. However, despite these findings, 70.6% of patients at the SOC visit reported “yes, definitely” if given the choice again to take this cancer therapy.

#### 4.1.5. WPAI: GH V2.0

Only four VOLUME-PRO patients reported being currently in paid employment during the study, making it difficult to interpret the results of the WPAI: GH V2.0.

#### 4.1.6. Safety

While medically important events were noted in approximately 30% of VOLUME patients taking nivolumab, it is important to note that events captured were not necessarily treatment related. For VOLUME, all medically important events were collected retrospectively and with no ability to discern cause and relation to nivolumab. Death while undergoing treatment occurred in <13% of patients, and for most was explained by the advanced stage of disease despite nivolumab initiation. While the primary objective of the VOLUME-PRO study was to assess the patients’ experience of nivolumab, the assessment of safety events was also of interest. In VOLUME-PRO, diarrhea and pruritis were most frequently reported, each with 3 (5.9%) incidents. Both events are noted as common side effects of nivolumab, and there were no new safety concerns identified through this study [42]. The proportion of patients reporting an SAE (13.7%) was similar to the risk of SAEs estimated in a meta-analysis of nivolumab trials (11.2%) [43]. The seven SAEs reported in VOLUME-PRO were unique and did not display a discernable pattern. It is likely that some were related to progressive disease.

### 4.2. Limitations

One limitation in this study was the variability of intra-site processes. Given the real-world study design of both VOLUME and VOLUME-PRO, processes between sites were not standardized. Inter-investigator variability across sites in the assessment of radiological responses was a potential source of bias in the interpretation of this endpoint. In addition, chart-based studies include the limited availability of treatment data, and may result in an underreporting of outcomes, particularly toxicity data, if a patient left the center or was not adequately followed-up, which could lead to missing or incomplete patient information. Additionally, the follow-up duration for VOLUME and VOLUME-PRO was quite minimal and may contribute to a lack of understanding of the true long-term effects of nivolumab over an extended period of time.

This study may underestimate the clinical benefit of nivolumab by under-representing the duration of treatment due to the definition of treatment discontinuation of 6 weeks without therapy used in this study. The definition of discontinuation may also overestimate the number of treatment lines, as some centers consider restarting treatment after more than 6 weeks of no infusion as the same line of therapy while in others this was considered a separate line of treatment. Creating a specific definition for treatment discontinuation allows for comparison across sites and countries and the results should be interpreted with this study’s definition in mind. Lastly, the study was conducted during the coronavirus (COVID-19) pandemic and as a result study recruitment ended earlier than expected. Aside from enrollment challenges caused by COVID-19, there were no formal analyses conducted to assess the impact of COVID-19 on follow-up assessment availability. However, the COVID-19 pandemic may have affected patients’ access to healthcare resourcing such as imaging and testing services. Therefore, the true effect of COVID-19 on the study results is unknown.

Lastly, clinical response assessment was defined based on physician assessment, which was not standardized in this real-world study. With the absence of specified response assessment criteria (i.e., RECIST, imaging) across sites, there is a possibility of misclassification due to intra-site variability. Physicians may overestimate responses to treatment compared to the RECIST criteria. However, no formal statistical assessments were conducted to determine misclassification within this real-world study.

## 5. Conclusions

The VOLUME and VOLUME-PRO studies provide a unique opportunity to observe the real-world experience of SCCHN patients receiving nivolumab across 23 different sites worldwide. Due to the real-world nature of this study, we were better able to capture data among patients who potentially had more comorbidities, were less fit, or may have more symptoms than those who would participate in controlled clinical trials. Our study designs allowed for a more realistic and comprehensive approach that ultimately garnered promising results that shed light on nivolumab treatment patterns and R/M SCCHN patient outcomes outside the context of clinical trials.

These studies are in alignment with the effectiveness and safety of nivolumab previously reported in the CheckMate 141 trial and other real-world studies of nivolumab in this population [6,44,45]. Further, the VOLUME-PRO study results underscore HRQoL benefits reported in CheckMate 141, where nivolumab did not decrease the HRQoL of patients. Our VOLUME-PRO results revealed that the majority of patients reported stability or improvement in their HRQoL domains over the 6 to 8 weeks from enrollment, though no formal hypothesis testing was conducted. In conclusion, this real-world multinational, multicenter, retrospective, and prospective study confirms the effectiveness and safety of nivolumab for patients with R/M SCCHN.

## Figures and Tables

**Table 1:** Patient demographic and clinical characteristics.

	VOLUME	VOLUME-PRO	Total
Characteristics	(*N* = 447)	(*N* = 51)	(*N* = 481)
	*n* (%)	*n* (%)	*n* (%)
Age			
Mean (SD)	63.2 (8.72)	63.2 (9.50)	63.2 (8.81)
Median (IQR)	64.0 (12.0)	64.0 (14.0)	64.0 (12.0)
Min; Max	35; 89	32; 77	32; 89
Sex			
Male	360 (80.5)	43 (84.3)	389 (80.9)
Female	87 (19.5)	8 (15.7)	92 (19.1)
Smoking status			
Current	109 (26.6)	12 (25.5)	117 (26.4)
Former	216 (52.7)	23 (48.9)	233 (52.6)
Never	85 (20.7)	12 (25.5)	93 (21.0)
ECOG ^1^			
0	86 (24.6)	13 (31.0)	94 (25.1)
1	205 (58.6)	23 (54.8)	218 (58.1)
2	50 (14.3)	4 (9.5)	53 (14.1)
3	8 (2.3)	2 (4.8)	9 (2.4)
4	1 (0.3)	0	1 (0.3)
Primary tumor location			
Oral cavity	122 (27.4)	17 (33.3)	132 (27.5)
Oropharynx	155 (34.8)	15 (29.4)	166 (34.6)
Larynx	63 (14.1)	12 (23.5)	73 (15.2)
Hypopharynx	42 (9.4)	1 (2.0)	42 (8.8)
Nasopharyngeal	11 (2.5)	0	11 (2.3)
Nasal cavity	3 (0.7)	1 (2.0)	3 (0.6)
Paranasal	1 (0.2)	1 (2.0)	2 (0.4)
Middle ear	1 (0.2)	0	1 (0.2)
Other ^2^	48 (10.8)	4 (7.8)	50 (10.4)
Metastasis location ^3^			
Lung	198 (45.7)	24 (48.0)	217 (46.5)
Bone	53 (12.2)	4 (8.0)	56 (12.0)
Liver	43 (9.9)	2 (4.0)	45 (9.6)
Mediastinum	39 (9.0)	7 (14.0)	43 (9.2)
Skin	18 (4.2)	0	18 (3.9)
Bone marrow	2 (0.5)	0	2 (0.4)
Other ^4^	198 (45.7)	23 (46.0)	215 (46.0)
PD-L1 prior to nivolumab start (% expression) ^5^			
n	67	14	78
Mean (SD)	21.4 (31.38)	10.8 (22.60)	19.3 (29.96)
Median (IQR)	5.0 (34.0)	1.5 (4.0)	2.5 (24.0)
Min; Max	0; 95	0; 75	0; 95
HPV status			
p16 positive	105 (51.7)	9 (47.4)	114 (52.3)
p16 negative	98 (48.3)	10 (52.6)	104 (47.7)
Prior lines of therapy ^6^			
1	269 (72.1)	27 (87.1)	285 (72.5)
2	78 (20.9)	2 (6.5)	80 (20.4)
≥3	26 (7.0)	2 (6.5)	28 (7.1)
Prior and concomitant anti-cancer therapies for SCCHN			
Any surgery	88 (19.7)	10 (19.6)	94 (19.5)
Any radiotherapy	229 (51.2)	18 (35.3)	239 (49.7)
Any cytotoxic drugs	377 (84.3)	32 (62.7)	397 (82.5)

ECOG: Eastern Cooperative Oncology Group; HPV: Human papillomavirus; IQR: Interquartile range; Max: maximum; Min: minimum; PD-L1: programmed death-ligand 1; SCCHN: squamous cell cancer of the head and neck; SD: standard deviation; TPS: tumor proportion score. ^1^ Only assessed for patients with an ECOG performance status assessment. ^2^ This includes, but is not limited to, sites such as esophagus, tonsil, sinus, unknown primary sites, or multi-site primary tumors. ^3^ Categories are not mutually exclusive. ^4^ This includes, but is not limited to, sites such as lymph nodes, adenopathy, or locoregional metastasis. ^5^ PD-L1 was assessed via TPS. ^6^ Only assessed for patients with at least one systemic cytotoxic drug.

**Table 2:** Nivolumab exposure.

Average dose ^1^ (mg per infusion)	
N	435
Mean (SD)	255.3 (90.23)
Median (IQR)	240.0 (36.0)
Min; Max	111; 480
Number of nivolumab treatment lines per patient ^2^	
N	447
Mean (SD)	1.1 (0.26)
Median (IQR)	1.0 (0.0)
Min; Max	1; 2
Action taken with nivolumab ^2^	*n* (%)
Dose increase	9 (2.0)
Dose reduction	3 (0.7)
Temporary interruption	77 (17.2)
Permanent interruption	202 (45.2)
Treatment restart ^3^	*n* (%)
Yes	69 (26.6)
No	190 (73.4)
Reason for drug discontinuation	*n* (%)
Progressive disease/lack of effectiveness	127 (62.9)
Death	28 (13.9)
Adverse event	17 (8.4)
Loss to follow-up	6 (3.0)
Patient withdrew consent	3 (1.5)
Insurance/economic reasons	0
Other	21 (10.4)
Permanent drug discontinuation due to any cause (including death) ^4^	
N (%)	434 (97.1)
Median time (months) to discontinuation (95% CI)	3.2 (2.8, 3.7)

CI: Confidence Interval; IQR: Interquartile range; Max: maximum; Min: minimum; SD: standard deviation. ^1^ Doses marked as ‘Other, specify’ were not considered in this derivation. ^2^ The categories are not mutually exclusive and are taken directly from case report form. ^3^ Percentages calculated using the number of patients with a temporary or permanent interruption of nivolumab. ^4^ Permanent drug discontinuation is defined as no infusion 6 weeks or more after the last infusion if the dose given is recorded as 3 mg/kg or 240 mg every 2 weeks, or 9 weeks or more if the dose given is recorded as 480 mg every 4 weeks. If nivolumab is restarted after the period mentioned, it should be considered as part of a new treatment line. Data for this field are derived from available data.

**Table 3:** Summary of PRO scores at enrollment and at SOC visit for VOLUME-PRO patients.

	VOLUME-PRO (*N* = 51)	
	Enrollment (Mean [SD])	SOC Visit (Mean [SD]) ^1^
**EORTC QLQ-C30 ^2^**		
N (%)	50 (98.0)	42 (82.4)
-Physical functioning score	70.8 (23.56)	69.7 (26.18)
-Role functioning score	67.7 (33.91)	68.3 (34.49)
-Emotional functioning score	76.6 (22.57)	78.2 (23.35)
-Cognitive functioning score	82.7 (19.33)	84.9 (17.58)
-Social functioning score *	70.3 (31.82)	76.4 (29.81)
-Fatigue score	39.3 (28.32)	39.9 (30.36)
-Nausea and vomiting score	13.0 (21.11)	11.9 (20.92)
-Pain score	30.3 (26.66)	29.0 (25.78)
-Dyspnea score	25.3 (30.54)	28.6 (33.39)
-Insomnia score	30.7 (30.00)	31.0 (32.42)
-Appetite loss score	31.3 (37.14)	31.0 (36.36)
-Constipation score	20.0 (30.12)	23.0 (31.66)
-Diarrhea score	9.3 (22.38)	11.9 (25.31)
-Financial difficulties score *	18.0 (27.94)	11.4 (23.11)
-Global health status/QoL score	64.2 (24.24)	61.9 (21.32)
**EORTC QLQ-H&N35 ^3^**		
N (%)	50 (98.0)	42 (82.4)
-Pain score	26.5 (23.00)	24.0 (23.08)
-Swallowing score	31.1 (31.63)	29.7 (32.33)
-Senses score	28.0 (36.18)	23.5 (32.61)
-Speech score	28.7 (28.40)	25.9 (28.27)
-Social eating score	34.7 (36.02)	31.0 (34.25)
-Social contact score	22.2 (28.25)	18.8 (26.23)
-Sexuality score	34.8 (39.70)	40.4 (38.09)
-Teeth score	23.8 (35.36)	16.3 (28.01)
-Open mouth score	34.0 (39.55)	33.3 (38.25)
-Dry mouth score	42.0 (33.54)	39.7 (35.49)
-Sticky saliva score	48.7 (36.40)	39.8 (40.29)
-Coughing score	34.0 (32.64)	31.0 (27.93)
-Feeling ill score	23.3 (30.30)	27.0 (32.29)
-Painkillers score	22.0 (15.95)	23.8 (15.24)
-Nutritional supplements score	14.0 (16.62)	15.1 (16.79)
-Feeding tube score	8.2 (14.48)	6.3 (13.25)
-Weight loss score	10.0 (15.43)	11.1 (15.90)
-Weight score	7.3 (13.95)	7.9 (14.37)
**EQ-5D-5L ^4^**		
N (%)	50 (98.0)	44 (86.3)
-EQ-5D index score	0.8 (0.21)	0.8 (0.23)
-EQ-5D-VAS score	67.9 (25.13)	70.1 (22.00)
**CTSQ ^5^**		
N (%)	49 (96.1)	42 (82.4)
-Expectations of therapy score	68.3 (23.90)	63.1 (23.86)
-Feelings about side effects score	60.6 (13.75)	64.7 (14.53)
-Satisfaction with therapy score	37.6 (10.67)	37.1 (13.15)
**WPAI: GH ^6^**		
N (%)	49 (96.1)	41 (80.4)
-Absenteeism score *	25.0 (50.00)	50.0 (57.74)
-Presenteeism score	6.7 (11.55)	0.0 (0.00)
-Work productivity loss score	100.0 (0.00)	100.0 (0.00)
-Activity impairment score	39.2 (29.99)	36.6 (26.89)

CTSQ: Cancer Therapy Satisfaction Questionnaire; EORTC QLQ-C30: European Organization for Research and Treatment of Cancer, Quality of Life Questionnaire-Core 30; EORTC QLQ-H&N35: European Organization for Research and Treatment of Cancer, Quality of Life Questionnaire-Head and Neck 35; EQ-5D-5L: 5-Level EQ-5D; HRQoL: health-related quality of life; QoL: quality of life; SD: standard deviation; SOC: standard of care; VAS: visual analog scale; WPAI:GH: Work Productivity and Activity Impairment Questionnaire: General Health. ^1^ SOC visit occurred for VOLUMEPRO patients approximately 6 to 8 weeks after the enrollment visit. ^2^ Higher scores for the EORTC QLQ-30 indicate a better QoL for functional scales and global health status. Lower scores for symptom scales represent a better QoL. ^3^ Higher scores indicate a better HRQoL for functional scales of the EORTC QLQ-H&N35 and global health status. Lower scores for symptom scales represent a better HRQoL. ^4^ For the EQ-5D-5L better health status is indicated by values closer to 1. The VAS score endpoints are labeled ‘Best imaginable health state’ and ‘Worst imaginable health state’, with numeric values of 100 and 0, respectively. ^5^ Higher CTSQ scores indicate better satisfaction. ^6^ WPAI scores are calculated as impairment percentages, and higher numbers indicate greater impairment and less productivity (i.e., worse outcomes). * Scores exceed the minimally important difference (EORTC QLQ-C30 = 5–10; WPAI = 7%).

## Data Availability

Data are available upon reasonable request if consistent with ethical approvals.

## Supplementary Materials

The following supporting information can be downloaded at: https://www.mdpi.com/article/10.3390/cancers15143552/s1, Figure S1: EQ-5D-5L dimension bar charts by visit type; Table S1: Subsequent anti-cancer therapy following nivolumab in VOLUME patients (*N* = 447); Table S2: Overall survival by age group (*N* = 447); Table S3: Overall Survival by ECOG performance status (*N* = 341); Table S4: Wilcoxon paired test for the PRO scores at Enrollment and SOC visit: VOLUME-PRO.
