# Supplementary material for: Phase 4 Multinational Multicenter Retrospective and Prospective Real-World Study of Nivolumab in Recurrent and Metastatic Squamous Cell Carcinoma of the Head and Neck
Source: Cancers (Basel). 2023 Jul 9;15(14):3552. doi: 10.3390/cancers15143552 (PMC10377225; doi:10.3390/cancers15143552)
Supplement: Supplementary file 1 [file cancers-15-03552-s001.zip › Supplementary Table S1.pdf]

cancers

Table S1: Subsequent Anti-cancer Therapy Following Nivolumab in VOLUME Patients (N=447)

| | **N (%)** |
|---|---|
| Any anti-cancer therapy | 140 (31.3) |
| Any surgery | 6 (4.3) |
| Any radiotherapy | 47 (33.6) |
| Any cytotoxic drugs | 104 (74.3) |
| Paclitaxel* | 64 (61.5) |
| Carboplatin* | 26 (25.0) |
| Cetuximab* | 24 (23.1) |
| Methotrexate* | 20 (19.2) |
| Docetaxel* | 18 (17.3) |
| 5-fluorouracil* | 8 (7.7) |
| Cisplatin* | 7 (6.7) |
| Other* | 23 (22.1) |

* Only assessed for patients with at least one cytotoxic drug

Percentages based on the number of patients with at least one post-nivolumab treatment and non-missing data.
