# Supplementary material for: Phase 4 Multinational Multicenter Retrospective and Prospective Real-World Study of Nivolumab in Recurrent and Metastatic Squamous Cell Carcinoma of the Head and Neck
Source: Cancers (Basel). 2023 Jul 9;15(14):3552. doi: 10.3390/cancers15143552 (PMC10377225; doi:10.3390/cancers15143552)
Supplement: Supplementary file 1 [file cancers-15-03552-s001.zip › Supplementary Table S2.pdf]

cancers

MDPI

Table S2: Overall Survival by Age Group (N=447)

	≤65 years old	>65 years old
Number of patients	263	184
Time to event (months) [a]		
Median (95% CI)	9.6 (8.2;10.8)	8.6 (7.3;13.7)
Range (with censored patients)	0.0-44.8	0.2-46.5
Event-free rate at		
6 months (95% CI)	63.3 (57.0; 68.9)	64.3 (56.7; 70.9)
9 months (95% CI)	52.0 (45.5; 58.0)	49.7 (42.0; 57.0)
1 year (95% CI)	39.0 (32.7; 45.2)	44.6 (36.9; 51.9)
2 years (95% CI)	24.6 (18.9; 30.7)	24.1 (16.9; 32.0)
3 years (95% CI)	18.6 (12.4; 25.8)	16.3 (9.0; 25.4)

CI: Confidence interval

[a] Kaplan-Meier estimations
