# Supplementary figures and images for: Phase 4 Multinational Multicenter Retrospective and Prospective Real-World Study of Nivolumab in Recurrent and Metastatic Squamous Cell Carcinoma of the Head and Neck
Source: Cancers (Basel). 2023 Jul 9;15(14):3552. doi: 10.3390/cancers15143552 (PMC10377225; doi:10.3390/cancers15143552)

1

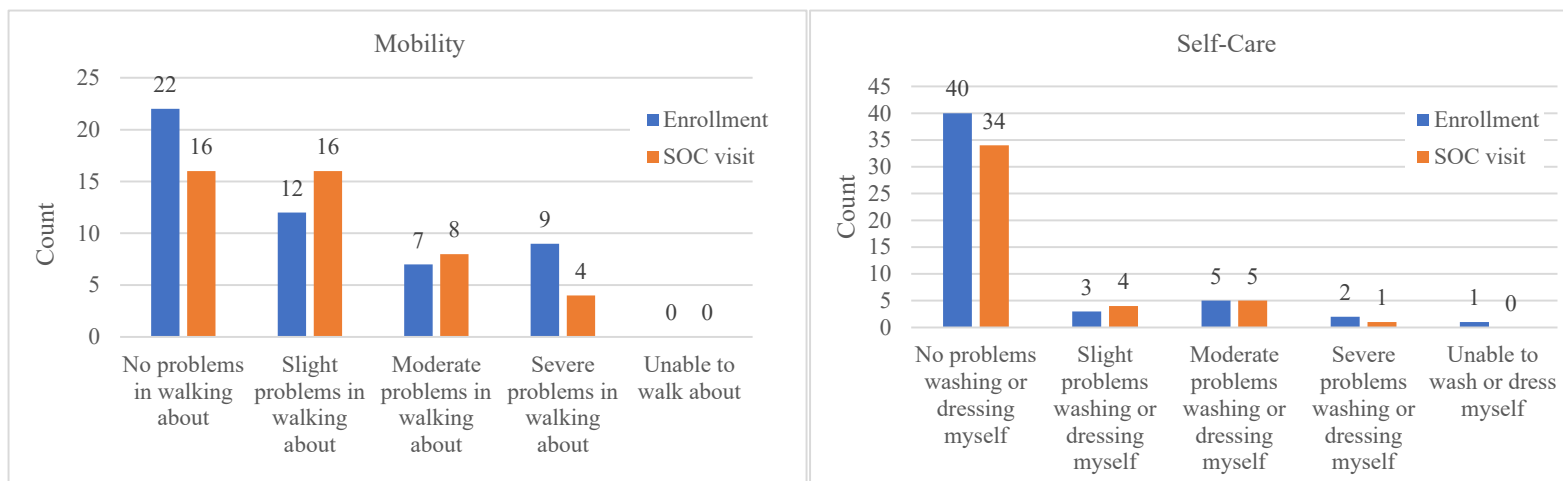

2

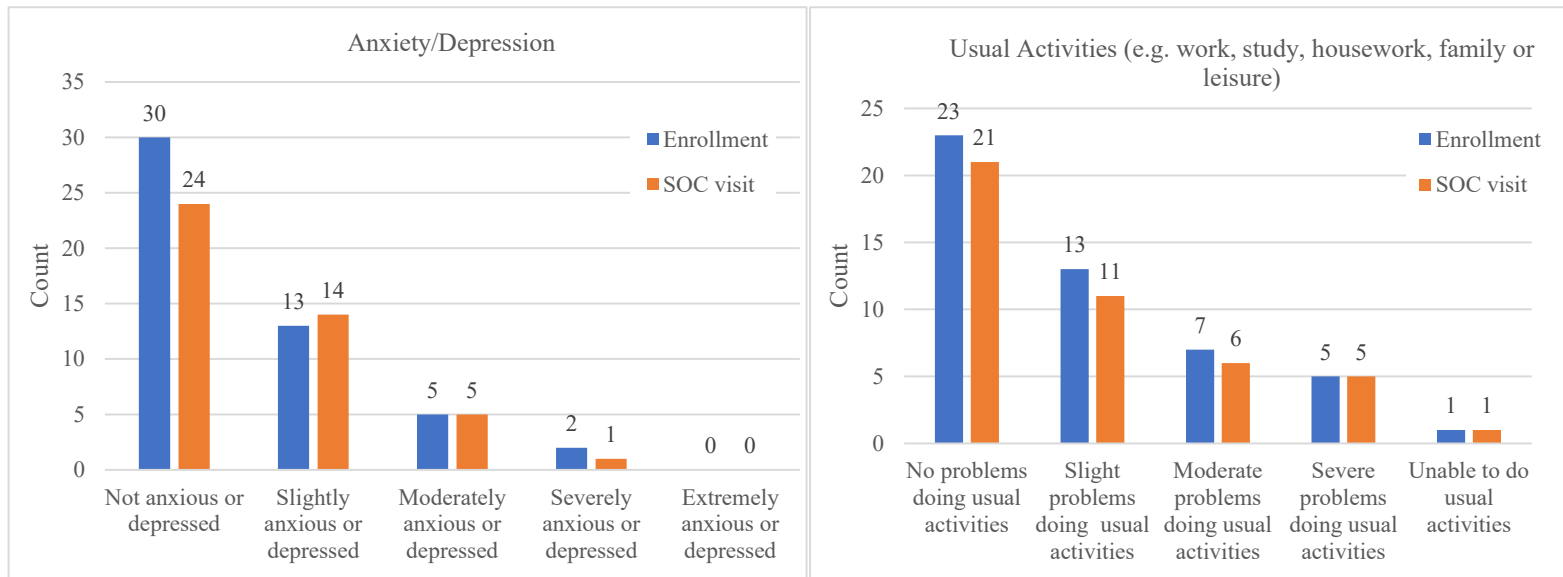

3

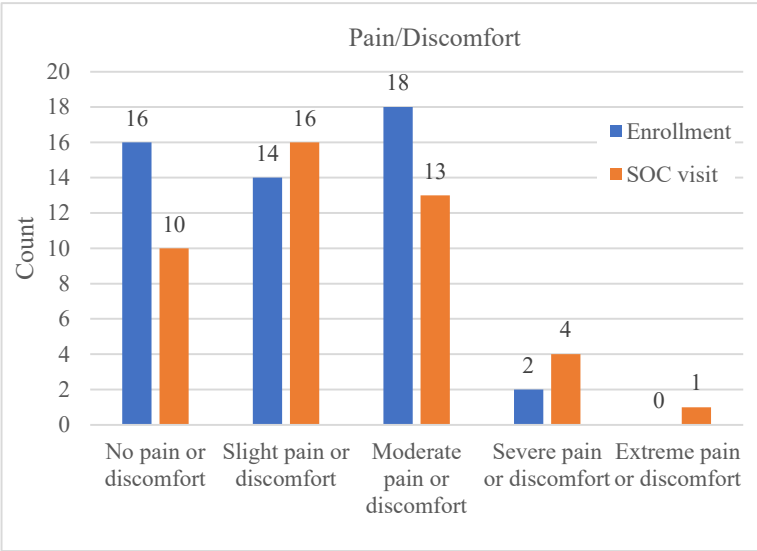

SOC: Standard of Care

Figure S1: EQ-5D-5L Dimension Bar Charts, by Visit Type

4  
5  
6

Supplement: Supplementary file 1 [file cancers-15-03552-s001.zip › Supplementary Figure S1.pdf]
